# Microbial Community Dynamics During the Non-filamentous Fungi Growth-Based Fermentation Process of *Miang*, a Traditional Fermented Tea of North Thailand and Their Product Characterizations

**DOI:** 10.3389/fmicb.2020.01515

**Published:** 2020-07-14

**Authors:** Kridsada Unban, Nuttapong Khatthongngam, Thanawat Pattananandecha, Chalermpong Saenjum, Kalidas Shetty, Chartchai Khanongnuch

**Affiliations:** ^1^Division of Biotechnology, School of Agro-Industry, Faculty of Agro-Industry, Chiang Mai University, Chiang Mai, Thailand; ^2^Department of Pharmaceutical Sciences, Faculty of Pharmacy, Chiang Mai University, Chiang Mai, Thailand; ^3^Global Institute of Food Security and International Agriculture (GIFSIA), Department of Plant Sciences, North Dakota State University, Fargo, ND, United States; ^4^Research Center for Multidisciplinary Approaches to Miang, Chiang Mai University, Chiang Mai, Thailand

**Keywords:** fermented tea, *Miang*, microbial community, antioxidants, fermentation

## Abstract

*Miang*, a traditional fermented tea leaf (*Camellia sinensis* var. *assamica*) consumed in northern Thailand, was simulated in laboratory conditions using non-filamentous fungi process (NFP) and microbial community was periodically investigated for over 6 months of fermentation by both culture-dependent and -independent techniques. The viable cell numbers of lactic acid bacteria (LAB), yeast, and *Bacillus* enumerated by the culture-dependent technique markedly surged over 3 days of initial fermentation and then smoothly declined by the end of fermentation. LAB were found as the main microbial population throughout the fermentation period followed by yeast and *Bacillus*. High-throughput sequencing of microbial community during fermentation revealed that *Firmicutes* (86.9–96.0%) and *Proteobacteria* (4.0–12.4%) were the dominant bacterial phyla, whereas *Ascomycota* was found to be the main fungal phylum with an abundance of over 99% in the fungal community. The dominant bacterial family was *Lactobacillaceae* (39.7–79.5%) followed by *Acetobacteraceae*, *Enterobacteriaceae*, *Bacillaceae*, *Aeromonadaceae*, *Staphylococcaceae*, *Moraxellaceae*, *Clostridiaceae*, *Exiguobacteraceae*, *Streptococcaceae*, and *Halomonadaceae*. Meanwhile, the main fungal family was *incertae sedis Saccharomycetales* (75.6–90.5%) followed by *Pichiaceae*, *Pleosporaceae*, *Botryosphaeriaceae*, *Davidiellaceae*, *Mycosphaerellaceae*, and *Saccharomycodaceae*. In addition, *Lactobacillus* (29.2–77.2%) and *Acetobacter* (3.8–22.8%), and the unicellular fungi, *Candida* (72.5–89.0%) and *Pichia* (8.1–14.9%), were the predominant genera during the fermentation process. The profiles of physical and chemical properties such as *Miang* texture, pH, organic acids, polysaccharide-degrading enzyme activities, and bioactive compounds have rationally indicated the microbial fermentation involvement. β-Mannanase and pectinase were assumed to be the key microbial enzymes involved in the *Miang* fermentation process. Total tannin and total polyphenol contents were relatively proportional to the antioxidant activity. Lactic acid and butyric acid reached maximum of 50.9 and 48.9 mg/g dry weight (dw) at 9 and 63 days of fermentation, respectively. This study provided essential information for deeper understanding of the *Miang* fermentation process based on the chemical and biological changes during production.

## Introduction

*Miang* is a traditionally consumed after-meal chewed food product from fermented Assam tea leaves (*Camellia sinensis* var. *assamica*) produced by naturally occurring bacteria and fungi, and has been used for hundreds of years in the northern part of Thailand. Traditional *Miang* is prepared by fermentation of steamed tea leaves in a container such as a bamboo basket without adding other nutritional substances for several days or up to a year (Khanongnuch et al., 2017). *Miang* fermentation processes can be categorized into two processes: filamentous fungi growth-based process (FFP) and non-filamentous fungi growth-based process (NFP) (Khanongnuch et al., 2017). The FFP type is processed by two main steps, where a bunch of steamed tea leaves in succession allows the growth of filamentous fungi under aerobic conditions for 5–10 days and then followed by the anaerobic fermentation at an ambient temperature for 3–5 days. Differing from the FFP process, the NFP type does not require the growth of initial filamentous fungi, where the steamed tea leaves are directly fermented under anaerobic conditions and kept at ambient temperature for 1–4 weeks to 3–12 months. Recently, nutritional biotransformation in both types of *Miang* was evaluated, and it was found that the quality of *Miang* is affected by the microflora involved, the quality of tea leaves, and the fermentation process, which are different depending on the production area (Unban et al., 2019). During the fermentation process, the nutritional components of steamed tea leaves such as proteins, carbohydrates, and lipids are utilized by various microorganisms derived from the surrounding environment through an enzyme-linked catalytic biotransformation process for producing the various metabolites such as organic acids, amino acids, and health-relevant bioactive metabolites. Through biotransformation of macronutrients in *Miang*, some additional beneficial bioactive compounds such as phenolic metabolites are expected, which have health-relevant benefits beyond basic nutrition as is well-known in green tea and tea-related products that include catechins and derivatives, gallic acids, and tannins (Kawakami et al., 1987; Huang et al., 2016; Zhu et al., 2020). Various kinds of microorganisms participate in *Miang* fermentation and produce unique flavors, odor, and tastes. It has been reported that *L. plantarum* is the dominant species in *Miang* and plays important roles during fermentation (Okada et al., 1986). Many studies have reported that lactic acid bacteria (LAB), including *Lactobacillus pentosus*, *Lactobacillus vaccinostercus*, *Enterococcus casseliflavus*, *Enterococcus camelliae*, *Lactobacillus thailandensis*, *Lactobacillus camelliae*, and *Pediococcus siamensis*, are involved in *Miang* fermentation (Sukontasing et al., 2007; Tanasupawat et al., 2007). Moreover, yeast analysis in *Miang* sample collected from 28 sampling sites including eight provinces in upper northern Thailand reported that *Candida ethanolica* is the dominant species (Kanpiengjai et al., 2016). Recently, the significant presence of endospore forming bacteria were detected from 40 *Miang* samples with the average number ranging from 40 to 45% of total bacterial counts in *Miang* samples (Unban et al., 2019). However, these studies were based on culture-dependent methods and were limited to identifying the microorganisms, which grow on specific nutrient media. This approach of using only the culturable microorganisms may not reflect the true microbial composition of *Miang*.

Recent advances in high-throughput sequencing technology by using Illumina HiSeq platform are changing the way to investigate the microbial communities where a large number of sequences can be analyzed at comparatively low cost, increasing the depth of sequencing for understanding the dynamics of microbial community (Loman et al., 2012). It has already been successfully used to elucidate the complex microbial community in fermented vegetable food, traditional fermented soybean food, and fermented tea leaves in Myanmar (Liang et al., 2018; Bo et al., 2020; Tan et al., 2020). This high-throughput sequencing technology was applied for the first time to investigate the dynamic changes in the bacterial and fungal communities in *Miang* fermentation. Therefore, in the current study, the bacterial and fungal community dynamics and changes in health-relevant bioactive compounds in *Miang* fermentation by the non-filamentous fungi-based process were carried out using 16S rRNA gene sequencing and ITS region sequencing using Illumina HiSeq platform to investigate relationships between bioactive compound and composition of the *Miang* microbial community during fermentation.

## Materials and Methods

### *Miang* Preparation and Sampling

The NFP *Miang* samples were prepared at a local *Miang* producing plant (Papae district, Chiang Mai, Thailand) following the traditional method as described previously (Khanongnuch et al., 2017). Briefly, fresh young Assam tea leaves (*C. sinensis* var. *assamica*) were steamed for 3 h. Immediately after steaming, the leaves were spread on a bamboo tray and allowed to cool for 3 h in an open space. The 100-g portions of steamed leaves were wrapped with 70% (v/v) ethanol surface-sterilized banana leaves and were tightly packed in polyethylene plastic bags (polyethylene bags, 17.7 × 25.4 cm) and then all samples were promptly transported in an ice box to laboratory. *Miang* samples were incubated in a dark chamber to allow natural fermentation at ambient temperature (28 ± 2°C) for 180 days. Periodically over the fermentation period, *Miang* samples were collected every 3 days for a month and a week for 180 days. Samples were kept at −20°C for testing in triplicate at each time point.

### Enumeration of Microorganism by Culture-Dependent Methods

Total numbers of viable bacterial and yeast cells in *Miang* samples were enumerated as described previously (Unban et al., 2019). Briefly, 50 g of *Miang* sample was mixed with 200 ml of sterile 0.85% (w/v) sodium chloride solution and homogenized by a Masticator homogenizer (IUL Instruments, Barcelona, Spain) for 10 min. The diluted samples from 10^–2^ to 10^–8^ were spread on nutrient agar (NA); deMan, Rogosa, and Sharpe (MRS) agar supplemented with 0.015% (w/v) bromocresol purple; and Sabouraud dextrose agar (SDA) supplemented with 100 mg/l chloramphenicol for enumeration of total viable bacteria, LAB, and yeast, respectively. The remaining diluted samples were incubated in a water bath at 80°C for 12 min, and then the proper diluted solutions were spread on NA for counting of endospore-forming bacteria presumptively assumed to be *Bacillus* sp. (Santana et al., 2008). The agar plates were incubated at 30°C for 3 days and the microbial numbers were calculated as colony-forming units (CFU) per gram of sample.

### Extraction of Genomic DNA

Five portions of each *Miang* sample were randomly collected from different parts of the whole sample and 1 g of each portion was extracted for total genomic DNA using DNeasy Power Soil Kit (Qiagen, Valencia, CA, United States), following the instruction of the manufacturer. DNA quality and quantity were assessed by ratios of 260/280 nm and 260/230 nm. Genomic DNA was stored at −20°C until further processing. A combination of five total genomic DNA extracts from *Miang* sample (100 μl each) was pooled in one 1.5-ml tube, mixed well, and used as final genomic DNA template for PCR amplification of both bacterial 16S rRNA and fungal ITS rRNA genes.

### Amplicon Sequencing of 16S rRNA Gene

The V3–V4 region of bacterial 16S rRNA gene was amplified using the primers 341-F (5′-CCT AYG GGR BGC ASC AG-3′) and 806-R (5′-GGA CTA CNN GGG TAT CTA AT-3′) with a specific barcode at the 5′ end. The PCR reactions were carried out with Phusion^®^ High-Fidelity PCR Master Mix (New England Biolabs, Beverly, MA, United States). Thermal cycling conditions used were as follows: 5 min initial denaturation at 95°C; 25 cycles of denaturation at 95°C (30 s); annealing at 56°C (30 s), elongation at 72°C (40 s); and final extension at 72°C for 10 min. The PCR products were separated by 2% (w/v) agarose gel electrophoresis. All PCR products were mixed and purified with the Qiagen Gel Extraction Kit (Qiagen, Germany). The sequenced libraries of bacterial 16S rRNA genes were generated for high-throughput sequencing using TruSeq^®^ DNA PCR-Free Sample Preparation Kit (Illumina, San Diego, CA, United States) following the manufacturer’s recommendations and index codes were added. The library quality was assessed on the Qubit@ 2.0 Fluorometer (Invitrogen, Thermo Scientific, CA, United States) and Agilent Bioanalyzer 2100 system (Agilent Technologies, Palo Alto, CA, United States). Then, the library was sequenced on an Illumina HiSeq2500 platform (Illumina, San Diego, CA, United States) by Novogene Bioinformatics Technology Co., Ltd. (Beijing, China).

### Amplicon Sequencing of ITS Region

The ITS rRNA gene of fungi was amplified with the forward primer ITS3 (5′-GCA TCG ATG AAG AAC GCA GC-3′) and the reverse primer ITS4 (5′-TCC TCC GCT TAT TGA TAT GC-3′) with a specific barcode at the 5′ end. The PCR reactions were carried out with Phusion High-Fidelity PCR Master Mix (New England Biolabs, Beverly, MA, United States). Thermal cycling conditions used were as follows: 5 min initial denaturation at 95°C; 25 cycles of denaturation at 95°C (30 s); annealing at 56°C (30 s), elongation at 72°C (40 s); and final extension at 72°C for 10 min. The PCR products were separated by 2% agarose gel electrophoresis. All PCR products were mixed and purified with the Qiagen Gel Extraction Kit (Qiagen, Germany). The sequenced libraries of fungal ITS rRNA genes were generated for high-throughput sequencing using TruSeq DNA PCR-Free Sample Preparation Kit (Illumina, San Diego, CA, United States) following the manufacturer’s recommendations, and index codes were added. The library quality was assessed on the Qubit@ 2.0 Fluorometer (Invitrogen, Thermo Scientific, CA, United States) and Agilent Bioanalyzer 2100 system (Agilent Technologies, Palo Alto, CA, United States). Then, the library was sequenced on an Illumina HiSeq2500 platform (Illumina, San Diego, CA, United States) by Novogene Bioinformatics Technology Co., Ltd. (Beijing, China).

### Bioinformatics

The raw sequencing reads obtained from Illumina platform were then merged using FLASH software (FLASH v1.2.7) (Magoč and Salzberg, 2011) and filtered with the QIIME software (Version 1.7) (Caporaso et al., 2010). After quality control, all quality filtered sequencing reads were clustered into operational taxonomic units (OTUs) with a threshold of 97% sequence similarity by utilizing UPARSE software (Version 7.0) (Edgar, 2013). Species classification of the processed bacterial OTU was performed using the Greengenes Database based on Ribosomal Database Project (RDP) classifier 2.2 algorithm (Desantis et al., 2006). The Unite Database was used on BLAST algorithm for fungal representative sequence, which was calculated by QIIME software (Kõljalg et al., 2013). The OTUs abundance information was normalized by utilizing a standard sequence number corresponding to the sample with the least sequences.

### Statistical Analysis

The alpha diversity indices, including Shannon-Weaver index (Shannon and Weaver, 1963), Cho1 index (Chao, 1987), and Good’s coverage values, were calculated by QIIME (Version 1.7)^1^ and displayed with R software (Version 2.15.3). Principal component analysis (PCA) based on the relative abundance of microbiota during the fermentation of *Miang* was performed using the FactoMineR package and ggplot2 package in R software (Version 2.15.3).

### pH and Total Acid

The pH of *Miang* samples was measured using a pH meter (OHAUS starter 2100 pH meter, Pine Brook, NJ, United States), and the total acid was determined by titration method as described by the Association of Official Analytical Chemists (AOAC, 2012). Briefly, 10 g of sample was suspended in 100 ml of sterile distilled water and homogenized for 10 min with a Masticator homogenizer (IUL Instruments, Barcelona, Spain) and then the supernatant was separated by centrifugation at 10,000 × *g* at 4°C for 10 min. The supernatant was used for pH measurement and total acid determination by the NaOH titration method.

### Organic Acid and Catechins Analysis

Eight major organic acids, including glucuronic acid, tartaric acid, lactic acid, acetic acid, citric acid, succinic acid, gallic acid, and butyric acid, in the *Miang* sample were determined using the method of Han et al. (2019) with slight modifications. Briefly, the analysis was conducted using an Agilent 1200 reversed-phase HPLC coupled with a UV detector equipped with Luna Omega Polar C18 UHPLC column (150 mm × 3.0 mm, 1.6-μm particle diameters, Phenomenex). The column was operated using 20 mM KH_2_PO_4_ as a mobile phase with a flow rate of 0.6 ml/min. The UV detection wavelength was set at 210 and 254 nm and the barbituric acid was used as the internal standard. All samples were analyzed in triplicate. The contents of extracted catechins and related compounds from *Miang* samples were analyzed by a reversed-phase HPLC using Agilent 1200 equipped with the Symmetry Shield RP18 column (4.6 mm × 250 mm, 5-μm particle diameters, Water Co., Ltd.). The column was operated at a flow rate of 1.0 ml/min using 10% acetonitrile in 0.1% acetic acid and H_2_O as mobile phase. The peaks were detected using a UV detector at 210 and 270 nm. All samples were measured in triplicate.

### Texture and Color Analysis

Texture profile analysis of the *Miang* sample was performed in terms of hardness using TA-XTplus Texture Analyser (Stable Micro Systems, Surrey, United Kingdom) in TPA mode. Quantitative evaluation of *Miang* surface color was measured by a portable tristimulus colorimeter (Minolta Chroma Meter CR-300, Osaka, Japan), and the result was presented by the L^∗^a^∗^b^∗^ color coordinate system (L^∗^ = lightness, a^∗^ = redness, and b^∗^ = yellowness). Five spots were randomly selected for color measurement on individual samples and presented as the average value.

### Determination of Polysaccharide-Degrading Enzymes

The polysaccharide-degrading enzyme activities (i.e., β-mannanase, cellulase, xylanase, amylase and pectinase) in *Miang* samples were investigated. β-Mannanase activity was estimated by measuring the amount of reducing sugars released by using the dinitrosalicylic acid (DNS) method (Miller, 1959). Briefly, the reaction mixture containing 0.125 ml of the desired dilution of enzyme and 0.125 ml of 0.5% (w/v) locust bean gum in 0.1 M sodium phosphate buffer (pH 6.5) was incubated at 37°C for 10 min. The reaction was stopped by addition of 0.25 ml of DNS (Sigma-Aldrich, St. Louis, MO, United States), then boiled for 10 min, and 2 ml of distilled water was added. The absorbance was measured at 540 nm. One unit of β-mannanase activity was defined as the amount of enzyme that liberated 1 μmol of reducing sugar per minute under the assay conditions. Cellulase, xylanase, pectinase, and amylase activities were also determined by the DNS method as described above, but 0.5% (w/v) of carboxymethyl cellulose (CMC), oat spelt xylan, pectin, and soluble starch in 0.1 M sodium phosphate buffer (pH 6.5) were specifically used as substrates for each enzyme.

### Sample Extraction for Bioactive Compound Analysis

*Miang* samples were dried at 50°C for 24 h in a vacuum drier (Binder VD 23, Germany) and the dried sample was powdered and sieved through 60 mesh (250 μm). A weight of 5 g of dry powdered *Miang* was extracted in 100 ml of 80% (v/v) acetone on an incubator shaker at 30°C with 250 rpm for 1 h. After extraction, the extract was filtered through Whatman filter paper (No. 1) to remove any suspended material. The solvent was completely removed from the filtrate at 40°C for 20 min by an EYELA N-1000 rotary evaporator (Tokyo Rika-kikai Co. Ltd., Japan). Dried *Miang* extract was dissolved with 20 ml of 80% (v/v) acetone, and the supernatant was used for the determination of total polyphenol content, total tannin, total flavonoid, and antioxidant activity based on DPPH free radical scavenging activity.

### Determination of Total Polyphenol Content

The total polyphenol content (TP) in *Miang* extract was determined spectrophotometrically according to a modified method of Eom et al. (2008), with Folin–Ciocalteu reagent. Briefly, 200 μl of the *Miang* extract sample was added into a test tube containing 200 μl of 2 M Folin–Ciocalteu reagent and vortexed. After adding 1.8 ml of deionized water, the reaction mixture was incubated at room temperature for 3 min. Then, 400 μl of 10% (w/v) sodium carbonate was added and vortexed. The final volume was adjusted to 4 ml by deionized water and incubated in the dark at room temperature for 1 h. The absorbance of blue coloration was measured at 725 nm using a spectrophotometer (Metertech SP8001, Taiwan) against a blank sample. Gallic acid was used as the standard and the results are expressed as milligrams of gallic acid equivalents (GAE) per gram of sample.

### Determination of Total Tannin Content

The total tannin content (TT) was determined by a modification of the Folin–Ciocalteu reagent according to Makkar et al. (1993) and using polyvinylpolypyrrolidone (PVPP) to separate tannins from other phenols. Briefly, 1 ml of the *Miang* extract sample was mixed with 1 ml of 10% (w/v) PVPP, vortexed, and kept at 4°C for 15 min. Then, the reaction mixture was centrifuged at 3,000 rpm for 10 min and the supernatant was collected. The remaining total phenol content of PVPP precipitated supernatant was measured with the Folin–Ciocalteu reagent and TT was estimated by using the following formula: TT = TP - PVPP precipitation. The results were expressed as milligrams of tannic acid equivalents (TAE) per gram of sample.

### Determination of Total Flavonoid Content

The content of total flavonoids (TF) in *Miang* extracts was determined spectrophotometrically according to the aluminum chloride colorimetric method (Eom et al., 2008). In brief, 100 μl of 10% (w/v) aluminum nitrate and 100 μl of 1 M potassium acetate were mixed with 500 μl of sample. Then, 3.3 ml of 80% (v/v) methanol was added to the reaction mixture and incubated for 40 min. The absorbance of the combination was read at 415 nm. Then, the absorbance values were converted to total flavonoid content expressed as milligrams of quercetin equivalents (QE) per gram of sample.

### Free Radical Scavenging Ability by DPPH Inhibition Assay

The antioxidant activity of the *Miang* extracts was analyzed according to the method reported by Braca et al. (2003) with minor modifications. This method is based on the scavenging activity of a stable 1,1-diphenyl-2-picrylhydrazyl (DPPH) free radical. In brief, 4 ml of 0.15 mM DPPH in ethanol solution was added to 1 ml of diluted extract sample (5, 2.5, 1.0, and 0.5 mg/ml) in deionized water and mixed vigorously. After 30 min of incubation in dark at room temperature, the absorbance was measured at 517 nm. The radical scavenging percentage was calculated against a blank using the following equation:

Inhibition(%)=(1-(B/A))×100

where *A* is the absorbance of the mixture without extract and *B* is the absorbance of the mixture containing the extract of the samples. The percentage of inhibition was calculated and a graphic of percentage of inhibition versus concentration was constructed. The DPPH scavenging activities are expressed as IC_50_ value (half maximal inhibitory concentration).

## Results and Discussion

### Microbial Changes During *Miang* Fermentation Process

**FIGURE 1:**
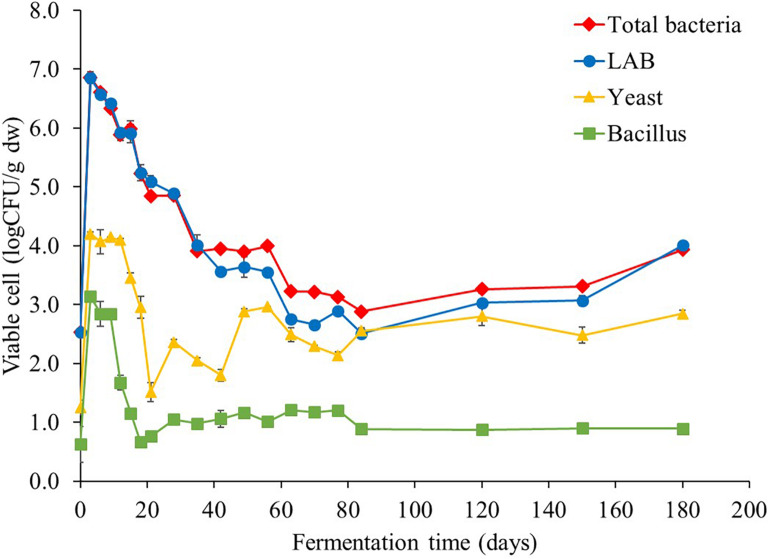
Changes in microbial population during *Miang* fermentation process. The values were derived from triplicate *Miang* samples and error bars represent standard deviations.

Viable cell counts associated with *Miang* fermentation process were periodically enumerated throughout the fermentation period (Figure 1). LAB were found as the major microbial population from initial stages to throughout the fermentation period following by yeast and *Bacillus*. At the beginning of fermentation, the initial LAB, yeast, and *Bacillus* were approximately 2.5, 1.2, and 0.6 logCFU/g dry weight (dw), respectively, and all microbial abundance rapidly increased to the highest levels of approximately 6.8, 4.2, and 3.1 logCFU/g dw, respectively, in just 3 days. However, LAB substantially decreased to 2.5 logCFU/g dw at day 84, after which, it became stable at approximately 2.5 logCFU/g dw until the end of fermentation. Meanwhile, yeast and *Bacillus* cell numbers stabilized during day 3 to day 12 around 4.1 and 3.0 logCFU/g dw, respectively, and then, both yeast and *Bacillus* numbers continuously declined to around 1.5 and 0.7 logCFU/g dw, respectively, by day 21. After which, yeast number gradually rose to around 2.5 logCFU/g dw by the end of fermentation, whereas *Bacillus* number remained steady approximately at 2.2 logCFU/g dw until the end of fermentation.

The results of viable cell number of all microbes markedly surged in the initial 3 days of fermentation, which indicated the log phase of microbial growth and the biomass was also doubled with every unit of time and linearly increased with time (Akerlund et al., 1995). After that, viable cell number of LAB tended to continuously decrease, while yeast and *Bacillus* tended to be stable during the entire fermentation period. This phenomenon is similar to the result of corresponding antagonistic relationship of yeasts and LAB in watery Kimchi (Jeong et al., 2013). Furthermore, mixed culture of microorganisms could affect various nutritional and biochemical changes in the fermentation process. The dynamics of fermentation in food matrix occurs by complex microbial processes via multiple mechanisms, which may have either positive, neutral, or negative impact on the fermentation process (Olanbiwoninu and Odunfa, 2018).

### Investigation on Bacterial and Fungal Community by Next-Generation Sequencing

**FIGURE 2:**
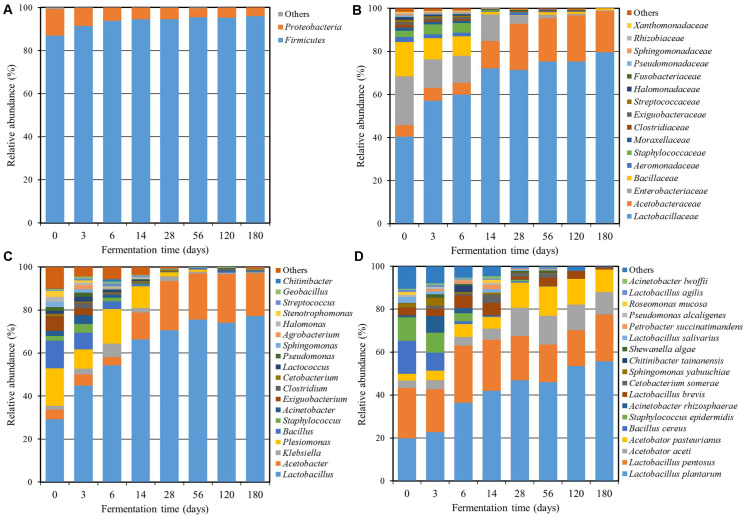
Relative abundance of the bacterial community at the phylum **(A)**, family **(B)**, genus **(C)**, and species **(D)** levels throughout the *Miang* fermentation period. The taxonomic abundance <0.1% was classified into “others.”

A total of 1,428,919 sequencing reads were obtained from a single run of 7 bacterial and 7 fungal PCR amplicons. After the removal of low-quality and chimera sequences, 350,050 bacterial and 633,740 fungal high-quality sequences with an average read length of approximately 428 bp (bacterial) and 290 bp (fungal) and an average of more than 87,074 reads (bacterial) and 90,534 reads (fungal) per sample were obtained, and their statistical diversities were calculated (Table 1). The values of OTUs and bacterial diversity indices clearly showed that the bacterial diversities in *Miang* increased during the early fermentation period and then decreased markedly after 3 days of fermentation. On the other hand, the fungal diversities also increased at the beginning of the fermentation period and were relatively constant during the entire fermentation period. The high quality of bacterial 16S rRNA gene and fungal ITS sequences was classified at the phylum, family, genus, and species level in order to investigate the microbial community changes during the *Miang* fermentation. The bacterial diversity was presented in Figure 2. A total of 8 bacterial phyla, 60 families, and 88 genera were identified. The main bacterial phyla were assigned to the phylum *Firmicutes* (86.9–96.0%) and *Proteobacteria* (4.0–12.4%) (Figure 2A). The dominant bacterial families were *Lactobacillaceae* (39.7–79.5%), *Acetobacteraceae* (5.2–21.4%), *Enterobacteriaceae* (0.4–22.3%), *Bacillaceae* (0.2–15.6%), *Aeromonadaceae* (0.0–2.4%), *Staphylococcaceae* (0.0–4.7%), *Moraxellaceae* (0.0–1.5%), *Clostridiaceae* (0.0–1.3%), *Exiguobacteraceae* (0.0–1.2%), *Streptococcaceae* (0.0–1.1%), and *Halomonadaceae* (0.0–1.1%) (Figure 2B). There were 18 dominant genera with an abundance over 1% including *Lactobacillus* (29.2–77.2%), *Acetobacter* (3.8–22.8%), *Klebsiella* (0.4–2.7%), *Plesiomonas* (0.1–17.5%), *Bacillus* (0.1–12.8%), *Exiguobacterium* (0.0–6.6%), *Staphylococcus* (0.0–4.1%), *Acinetobacter* (0.0–4.1%), *Stenotrophomonas* (0.0–2.6%), *Clostridium* (0.0–2.5%), *Sphingomonas* (0.0–2.5%), *Lactococcus* (0.0–2.2%), *Halomonas* (0.0–2.3%), *Pseudomonas* (0.0–1.9%), *Agrobacterium* (0.0–1.7%), *Streptococcus* (0.0–1.6%), *Chitinibacter* (0.0–1.2%), and *Cetobacterium* (0.0–1.0%) (Figure 2C). During the early fermentation period (day 3 and 6), the predominant bacteria were *Lactobacillus* followed by *Acetobacter*, *Klebsiella*, *Plesiomonas*, *Bacillus*, and *Staphylococcus*. However, the diverse bacterial community was replaced by *Lactobacillus* and *Acetobacter* as the fermentation progressed. The sequencing reads were further classified at the species level (Figure 2D). During early fermentation period (day 3), *Lactobacillus plantarum* (22.8%), *L. pentosus* (20.2%), *Acetobacter aceti* (4.1%), *Acetobacter pasteurianus* (4.5%), and *Bacillus cereus* (8.4%) were dominant. The observation was in agreement with results reported by Chaikaew et al. (2017), and the present study found that *L. plantarum* is the predominant species in *Miang*. It was also reported that *Lactobacillus* sp. occurred dominantly in various fermented tea products (Bo et al., 2020; Zhu et al., 2020).

**TABLE 1:** Summary of the sequencing data sets and statistical analysis of *Miang* fermentation samples.

Fermentation time (day)		Total reads	High-quality reads	Average read length (bp)	OTUs	Shannon-Weaver index	Choa1 index	Good’s coverage
Bacteria	0	114,475	58,426	429	98	2.07	132.42	99.8
	3	107,486	61,330	428	170	2.24	145.67	99.8
	6	105,370	49,144	429	120	1.82	117.33	99.5
	14	110,773	40,244	429	112	1.84	125.00	99.8
	28	111,566	61,195	429	83	1.54	118.30	99.6
	56	103,882	55,029	427	71	1.26	106.13	99.9
	120	86,185	29,676	428	54	1.04	30.87	99.7
	180	107,874	53,432	429	31	1.08	47.75	99.5
Fungi	0	102,342	94,754	291	124	2.23	157.61	99.7
	3	92,703	84,214	291	166	2.52	165.00	99.5
	6	99,702	91,718	290	172	2.56	175.12	99.6
	14	101,280	92,170	293	191	2.62	188.51	99.4
	28	104,960	95,308	291	183	2.30	186.03	99.8
	56	90,014	81,808	291	149	2.37	219.00	99.7
	120	108,354	98,302	289	145	2.18	154.37	99.8
	180	98,770	90,220	288	97	1.26	104.23	99.5

**FIGURE 3:**
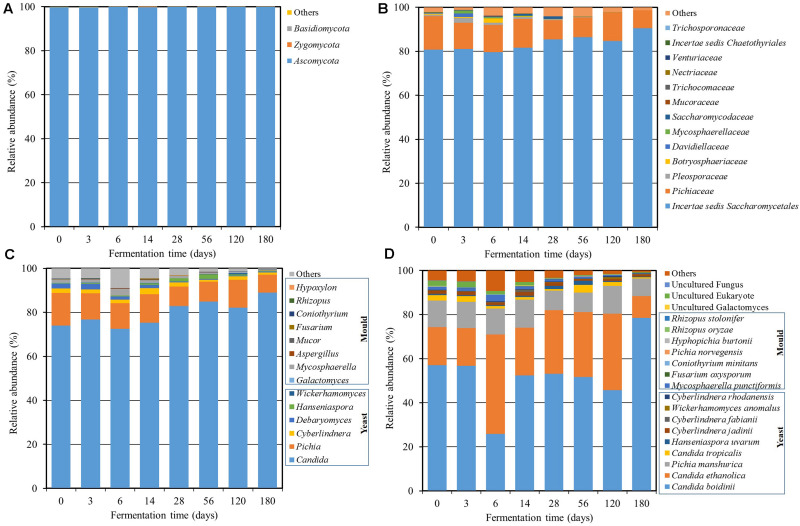
Relative abundance of the fungal community at the phylum **(A)**, family **(B)**, genus **(C)**, and species **(D)** levels throughout the *Miang* fermentation period. The taxonomic abundance <0.1% was classified into “others.”

In case of fungi, the fungal diversity was demonstrated in Figure 3. A total of 3 fungal phyla, 56 families, and 75 genera were found using amplicon sequencing. *Ascomycota* was the main fungal phylum with an abundance of over 99% in the community (Figure 3A). The dominant fungal family was incertae sedis *Saccharomycetales* (75.6–90.5%) followed by *Pichiaceae* (8.1–14.9%), *Pleosporaceae* (0.0–2.2%), *Botryosphaeriaceae* (0.0–1.9%), *Davidiellaceae* (0.0–1.7%), *Mycosphaerellaceae* (0.0–1.3%), and *Saccharomycodaceae* (0.0–1.1%) (Figure 3B). There were six dominant genera with an abundance over 1% including *Candida* (72.5–89.0%), *Pichia* (8.1–14.9%), *Mycosphaerella* (0.3–2.9%), *Cyberlindnera* (0.9–2.8%), *Debaryomyces* (0.3–2.4%), and *Hanseniaspora* (0.3–1.9%) (Figure 3C). It was found that the genus level consisted of diverse groups during the initial fermentation period. After that, *Candida* and *Pichia* increased as the dominant genera with fermentation time. The sequencing reads were further classified at the species level (Figure 3D) and found that *Candida boidinii* (25.9–78.6%), *C. ethanolica* (9.8–45.1%), and *Pichia manshurica* (8.0–12.7%) were dominant species and their communities were relatively stable. The result was in agreement with a previous report (Kanpiengjai et al., 2016), which found that *C. ethanolica* was the dominant species in the *Miang* sample collected from 28 sampling sites in upper northern Thailand. The genera *Candida* and *Aspergillus* were also found in some other types of fermented tea product, such as Fu brick tea and La phet (Li et al., 2017; Bo et al., 2020). Kanpiengjai et al. (2016) also suggested that yeast was involved in different profiles of chemical composition, flavors, qualities, and other unique characteristics of *Miang*. This included unique aroma constituents such as acetic acid and 4-ethylphenol. The secondary alcohols such as 2-butanol, 2-heptanol, (E)-4-hepten-2-ol, and 1-octen-3-ol, as well as aromatic alcohols such as benzyl alcohol and 2-phenylethanol were also found.

**FIGURE 4:**
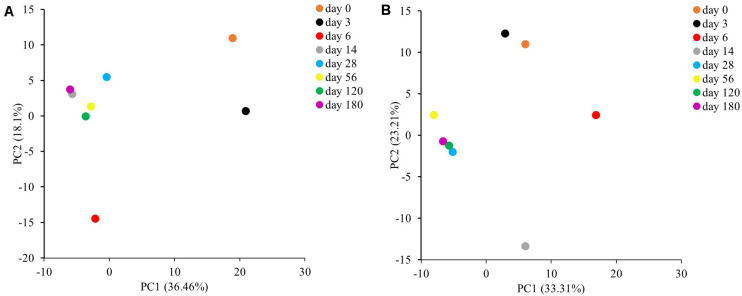
Principal component analysis of bacterial **(A)** and fungal **(B)** communities in sample during the *Miang* fermentation process.

In addition, to understand the variability among bacterial and fungal community structure during the fermentation process, PCA was performed (Figure 4). PCA based on bacterial 16S rRNA sequence data with PC1 and PC2 axes explained 36.46 and 18.10% of the variation in bacterial community differentiation, respectively. Meanwhile, PCA based on fungal ITS sequence data with PC1 and PC2 axes explained 33.31 and 23.21% of the variation in fungal community differentiation, respectively. The results suggest that the bacterial and fungal community structure during the *Miang* fermentation were changed significantly (*p* < 0.05) at the early stage of fermentation. Then, bacterial and fungal communities were clustered after 14 and 28 days of fermentation, respectively, indicating that microbial diversity changed slowly after these periods.

### Physico-Chemical Changes During Fermentation

**FIGURE 5:**
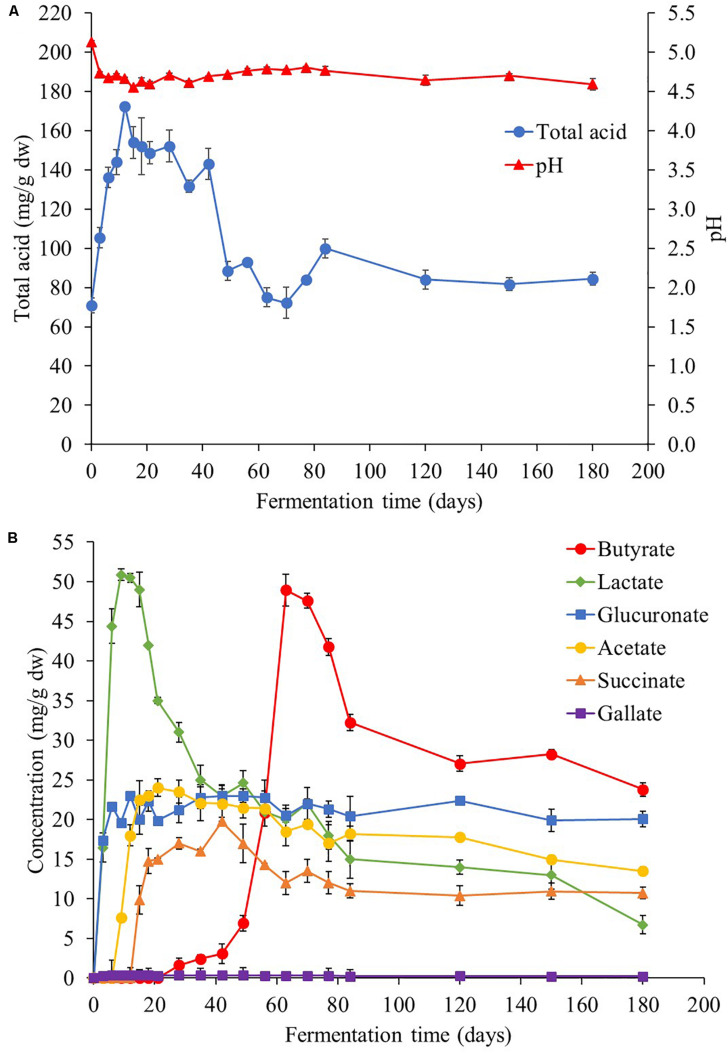
Total acidity and pH **(A)** and organic acid profile **(B)** during the *Miang* fermentation process. The values were derived from triplicate *Miang* samples and error bars represent standard deviations.

According to Figure 5A, the initial pH value of *Miang* was approximately 5.0 and decreased rapidly to around 4.7 at day 3. Then, the pH values became stable in the range of 4.5 to 4.7 until the end of fermentation. The organic acids from NFP *Miang* samples were extracted and analyzed by HPLC and various organic acids such as glucuronic acid, tartaric acid, lactic acid, acetic acid, citric acid, succinic acid, gallic acid, and butyric acid were detected as presented in Figure 5B. Inconsistent with the pH values, the total acid content of the *Miang* sample rapidly increased to the maximum approximately 0.17 g/g dw after 12 days of fermentation and remained steady with slightly fluctuation until 40 days and then slowly decreased to approximately 0.08 g/g dw at the end of fermentation. Decrease of pH values and increase of total acid content in the beginning stage should be the result of acid producing by microorganisms such as LAB (Xiao et al., 2015) and yeast (Mugula et al., 2003; Narvhus and Gadaga, 2003; Arroyo-López et al., 2008). However, inconsistency between the pH and total acid content detected might be attributed to some buffering effects of the fermentation broth (Neffe-Skocińska et al., 2017). Furthermore, based on details of organic acid species presented in Figure 5B, at the beginning of fermentation, lactic and glucuronic acids were initially present in the sample and lactic acid was found as the main organic acid in the first 4 weeks. Acetic acid and succinic acid were later detected at 7 and 14 days, respectively. Regarding the pH changes described previously, the prompt increase of lactic acid and glucuronic acid during the early fermentation period may correlate well with the decrease in pH (Figure 5B). The amount of lactic acid observed was nearly two times higher than other acids in the early stage and reached a maximum concentration of around 50.9 mg/g dw at day 9 of fermentation, which was also correlated with the maximum growth of LAB population presented in NFP *Miang* samples described previously (Figure 1). Regarding the increase of glucuronic acid to a maximum of about 20 mg/g dw at day 9, it has remained mostly stable until the end of fermentation. The well-known benefit of glucuronic acid is its detoxifying action through conjugation, which is also considered to be the important key component found in Kombucha tea (Jayabalan et al., 2007; Vina et al., 2013). Succinic acid is a by-product of yeast metabolism during fermentation (Tasev et al., 2016), increased maximum at 42-day and then tended to be stable to the end of fermentation. Interestingly, butyric acid was found at day 28 and increased rapidly to maximum concentration of 48.9 mg/g dw at 63 days of fermentation. Butyric acid is normally produced via glycolysis pathway by obligatory anaerobic spore-forming bacteria mainly and belong to the genus *Clostridium* (Jha et al., 2014). These bacterial group are able to oxidize sugar, and occasionally amylose and pectin to pyruvate and then to butyric acid (Ciani et al., 2008). The possible way of the *Clostridium* sp. growth is due to the likely decrease of oxygen concentration in the middle stage of *Miang* fermentation, which possibly occurred by oxygen consumption by aerobic bacteria at the early stage. Moreover, there is a growing number of reports describing the potential role of butyric acid related to treatment of hemoglobinopathies, cancer, and gastrointestinal diseases (Rephaeli et al., 2000; Encarnação et al., 2015). Further, in this study, gallic acid appeared throughout the fermentation period but a very low amount was detected.

**FIGURE 6:**
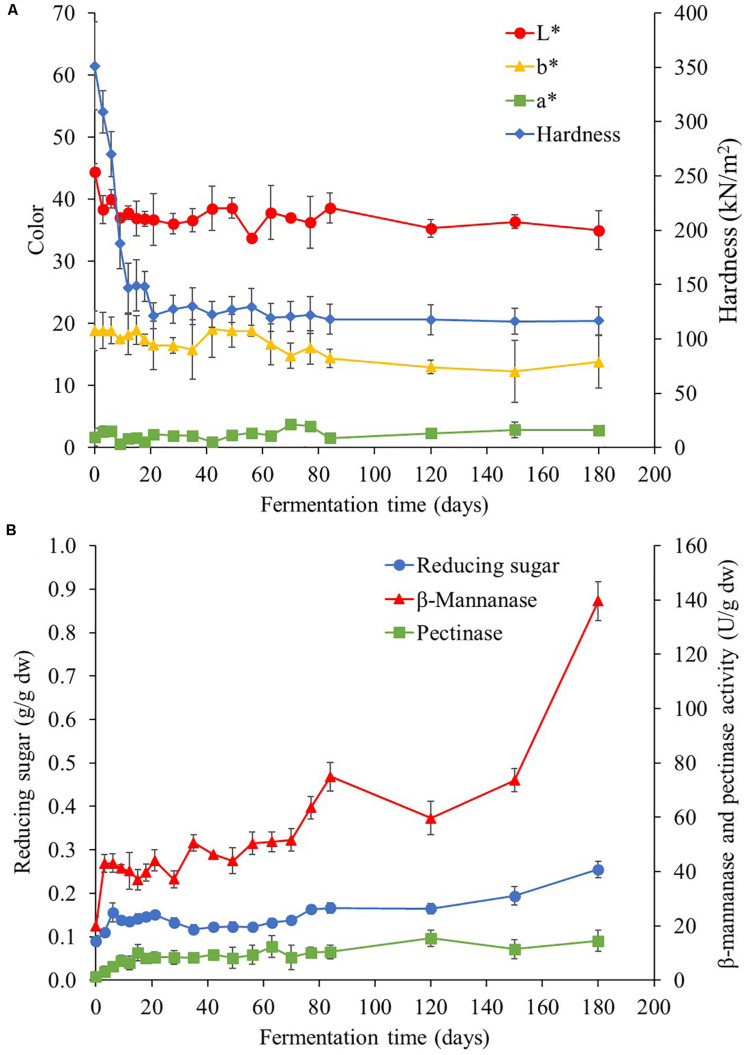
Changes in hardness and color **(A)** and reducing sugar and enzyme activity **(B)** during the *Miang* fermentation process. The values were derived from triplicate *Miang* samples and error bars represent standard deviations.

Texture analysis was measured in terms of hardness (Figure 6A). At the beginning, the hardness of *Miang* was at 3.5 × 10^5^ N/m^2^ and then sharply declined to 1.5 × 10^5^ N/m^2^ after 12 days of fermentation. Subsequently, hardness of *Miang* remained constant at about 1.2 × 10^5^ N/m^2^ until the end of fermentation. Decreasing hardness in the early stage of fermentation was potentially due to β-mannanase activity (Chauhan et al., 2012). This could result in softness of steamed tea leaves due to breakdown of hemicellulose leading to softness (Mcdougall et al., 1993; Sharma et al., 1999). Beside the change of hardness, the color changes are also presented in Figure 6A, where the L^∗^ value represents the degree of lightness, and the higher it is, the lighter the color. The a^∗^ value indicates redness when positive and greenness when negative, while the b^∗^ value reflects yellowness when positive and blueness when negative. For L^∗^ values, it significantly (*p* < 0.05) decreased from 44 at the beginning of fermentation to 38 at day 3 and then slowly decreased to 34 at the end of fermentation. For a^∗^ and b^∗^ values, the results indicated lack of significant change (*p* < 0.05) during the fermentation process. According to the result, it was found that only lightness had decreased. This might be caused by the oxidation of catechins to form larger molecules and non-volatile compounds such as theaflavins and thearubigins (Pou, 2016). Generally, the color of tea leaves is attributed to the phenolic compounds that they contain (Chaturvedula and Prakash, 2011) and to the oxidative enzymes (polyphenol oxidase), which could transform some phenolic compounds to phenolic pigments resulting in natural browning reaction (Kim et al., 2011).

### β-Mannanase and Pectinase Activity Profiles

Various polysaccharide-degrading enzyme activities such as cellulases, β-mannanase, xylanase, amylase, and pectinase were investigated during the *Miang* fermentation process, but β-mannanase and pectinase were only two polysaccharide-degrading enzymes found in NFP *Miang* samples. At the early stage of fermentation, 19 U/g dw of β-mannanase activity was detected and this increased to 43 U/g dw at day 3. Subsequently, the trend was rather stable until day 28 and reached up to 140 U/g dw at the end of fermentation. However, very low amount of pectinase activity (8.0 U/g dw) was detected in the early stage of fermentation and was continually stable until 180 days of fermentation. Changes in β-mannanase and pectinase activities corresponded to the decrease of hardness at the early stage of fermentation (Figure 6B). In correlation to the enzyme activity, the initial reducing sugar content was 0.09 g/g dw and then quickly increased to 0.15 g/g dw on day 6. After that, it continuously increased to 0.25 g/g dw by the end of fermentation (Figure 6B). Tea leaves consist of 16.2% cellulose, 68.2% hemicellulose, and 18.8% lignin (Rahman et al., 2017). These compositions could be degraded by enzymatic process of microorganisms, resulting in the released reducing sugar during the fermentation process. This phenomenon might have been caused by high demand of nutrition for microbial growth and reproduction in the log phase (Akerlund et al., 1995). Moreover, the increase of reducing sugar in fermenting tea leaves might be the result of the hydrolysis of polysaccharides by β-mannanase, which was the main enzyme activity detected in the *Miang* sample. The detectable polysaccharide hydrolyzing enzymes such as β-mannanase and pectinase coincide with the rapid growth of the main microbial community such as LAB, which is normally unable to produce those two main enzymes, which therefore demonstrates the synergistic relationship with the other microbial communities during *Miang* fermentation. Potentially, β-mannanase activity is expected to be produced by the *Bacillus* spp. as these are found in high numbers and based on previous reports, suggesting the capability of β-mannanase production by various Bacilli (Khanongnuch et al., 1998; Zhang et al., 2000; Dhawan and Kaur, 2007; Chauhan et al., 2012). In a similar way, pectinase is possibly from either yeast or *Bacillus* spp., which was also found in high numbers during *Miang* fermentation. Therefore, the LAB utilize hydrolysis products from these two enzymes in the metabolic pathway through the succession process and finally produce lactic acid as the final product.

### Profiles of Bioactive Compounds and Antioxidant Activity

**FIGURE 7:**
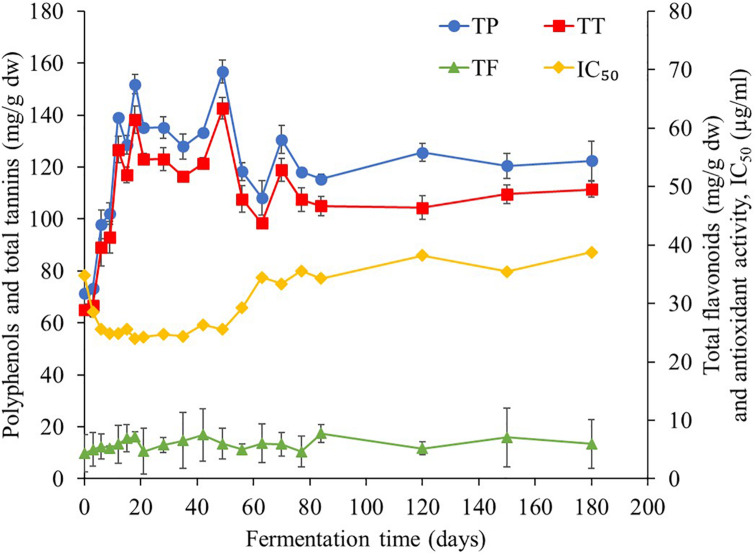
Changes in total polyphenol, total tannin, total flavonoids, and antioxidant activity during the *Miang* fermentation process. The values were derived from triplicate *Miang* samples and error bars represent standard deviations.

**FIGURE 8:**
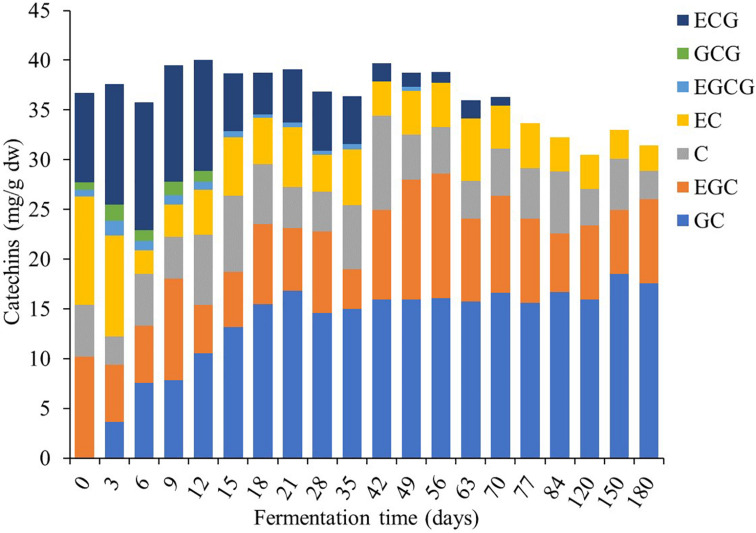
Changes in catechins during the *Miang* fermentation process; (+)-gallocatechin (GC), (−)-epigallocatechin (EGC), (+)-catechin (C), (−)-epicatechin (EC), (−)-epigallocatechin gallate (EGCG), (+)-gallocatechin gallate (GCG), and (−)-epicatechin gallate (ECG).

This study found that TP contents were the major bioactive compounds in *Miang* during the fermentation period (Figure 7) followed by TT and the TF. At the initial fermentation period, TP and TT contents increased to 151 and 138 mg/g dw, respectively, by day 18. After that, their trend was rather constant until 56 days of fermentation, following which contents decreased to 108 and 98 mg/g dw, respectively, on day 70 and then remained steady until the end of fermentation. For antioxidant activity, IC_50_ value tended to decrease from 34 to 25 μg/ml from day 0 to day 6 and then remained steady until 56 days of fermentation and subsequently increased back to 34 μg/ml on day 70. After this stage, it became stable until the end of fermentation. It was observed that after 70 days of fermentation, antioxidant activity decreased due to the decreasing of TP and TT. Change of bioactive compounds may be due to the ability of microorganisms to oxidize phenolic compounds of tea, leading to considerable loss of catechins and formation of theaflavins, thearubigins, theabrownins, and gallic acid (Kosińska and Andlauer, 2014). In addition, a previous report suggested that the yeast community mainly in the *Candida* sp. group was assumed to be associated with changes in the polyphenol and flavonoid contents in Kombucha (Chakravorty et al., 2016). Kanpiengjai et al. (2016) suggested that tannase-producing yeasts may be involved in the formation of catechin and their derivatives in *Miang* production. From the results of this study, it could be suggested that the fermentation of *Miang* between 18 and 56 days of fermentation is the proper fermentation time for obtaining high antioxidant activity from the metabolic process. The major polyphenolic compounds of tea leaves are catechins (flavan-3-ols), which are efficient free radical scavengers due to their one-electron reduction potential. Along the *Miang* fermentation process, catechins were changed and the content of catechins during *Miang* fermentation is shown in Figure 8. Overall, total catechins slightly decreased from 37.8 to 31.5 mg/g dw at the end of fermentation. GC was produced and increased from 3.6 mg/g dw at day 3 to 16.8 mg/g dw at day 21 and then stable until the end of the fermentation process. After 12 days of fermentation, ECG, GCG, and EGCG began to decrease and were not detected at 77 days of fermentation, which is the end of process. It is assumed that ECG, GCG, and EGCG were converted to their corresponding catechin EC, GC, and EGC, respectively. Biotransformation of ECG, GCG, and EGCG to EC, GC, and EGC by microbial enzymes in *Miang* could be the main reason for the increased concentration of EC, GC, and EGC. These trends correspond to previous studies of tea catechin degradation during the microbial fermentation in fermented tea products (Noh et al., 2014; Li et al., 2018).

## Conclusion

This study successfully evaluated the profiles of microbial communities during the fermentation of NFP *Miang* via culture-dependent investigation and targeted culture-independent method using high-throughput sequencing. *Lactobacillus* was clearly found as the most dominant bacterial group followed by *Acetobacter*, while *Candida* and *Pichia* were found as the highest dominant population of fungi. Microbial changes also directly affect the physical and chemical properties of NFP *Miang* product, which were revealed by the pH values and organic acid profiles. The most interesting organic acid, butyric acid, was also detected in NFP *Miang* product. Moreover, the health-relevant bioactive compounds and antioxidant activity reached a maximum value by days 18–56 of fermentation, which might be the most suitable stage to harvest and use *Miang* product for consumption. The results provide the basis to understand the mechanism of traditional fermented food, which is useful and essential for improving the industrial production of *Miang*. Further studies and future analyses of the association between the microorganisms and bioactive compounds that participate in *Miang* fermentation will help to advance knowledge for understanding the function of microorganisms in *Miang*-based traditional fermented foods, which can be targeted for applications and wider use.

## Data Availability Statement

The microbial metagenomic datasets generated for this study can be found in the Sequence Read Archive (SRA) at the NCBI under the BioProject under accession number PRJNA610188. The access link: http://www.ncbi.nlm.nih.gov/bioproject/610188.

## Author Contributions

KU, KS, and CK: conceptualization, writing—review and editing. KU and CK: methodology, writing—original draft preparation. KU and NK: formal analysis. KU, NK, TP, CS, and CK: investigation. KU: visualization. CK: supervision. All authors read and approved the final manuscript.

## Conflict of Interest

The authors declare that the research was conducted in the absence of any commercial or financial relationships that could be construed as a potential conflict of interest.

## Footnotes

1 http://qiime.org/tutorials/tutorial.html

## References

[B1] Akerlund T. Nordström K. Bernander R. (1995). Analysis of cell size and DNA content in exponentially growing and stationary-phase batch cultures of *Escherichia coli*. *J. Bacteriol.* 177 6791–6797. 10.1128/jb.177.23.6791-6797.1995 7592469 PMC177544

[B2] AOAC (2012). *Official Method of Analysis*, 18th Edn Washington DC: Association of official analytical chemists.

[B3] Arroyo-López F. Querol A. Bautista-Gallego J. Garrido-Fernández A. (2008). Role of yeasts in table olive production. *Int. J. Food Microbiol.* 128 189–196. 10.1016/j.ijfoodmicro.2008.08.018 18835502

[B4] Bo B. Kim S. A. Han N. S. (2020). Bacterial and fungal diversity in Laphet, traditional fermented tea leaves in Myanmar, analyzed by culturing, DNA amplicon-based sequencing, and PCR-DGGE methods. *Int. J. Food Microbiol.* 320:108508. 10.1016/j.ijfoodmicro.2020.108508 31986350

[B5] Braca A. Fico G. Morelli I. De Simone F. Tomè F. De Tommasi N. (2003). Antioxidant and free radical scavenging activity of flavonol glycosides from different Aconitum species. *J. Ethnopharmacol.* 86 63–67. 10.1016/S0378-8741(03)00043-6 12686443

[B6] Caporaso J. G. Kuczynski J. Stombaugh J. Bittinger K. Bushman F. D. Costello E. K. (2010). QIIME allows analysis of high-throughput community sequencing data. *Nat. Methods* 7 335–336. 10.1038/nmeth.f.303 20383131 PMC3156573

[B7] Chaikaew S. Baipong S. Sone T. Kanpiengjai A. Chui-Chai N. Asano K. (2017). Diversity of lactic acid bacteria from Miang, a traditional fermented tea leaf in northern Thailand and their tannin-tolerant ability in tea extract. *J. Microbiol.* 55 720–729. 10.1007/s12275-017-7195-8 28865074

[B8] Chakravorty S. Bhattacharya S. Chatzinotas A. Chakraborty W. Bhattacharya D. Gachhui R. (2016). Kombucha tea fermentation: microbial and biochemical dynamics. *Int. J. Food Microbiol.* 220 63–72. 10.1016/j.ijfoodmicro.2015.12.015 26796581

[B9] Chao A. (1987). Estimating the population size for capture-recapture data with unequal catchability. *Biometrics* 43 783–791. 10.2307/2531532 3427163

[B10] Chaturvedula V. S. P. Prakash I. (2011). The aroma, taste, color and bioactive constituents of tea. *J. Med. Plants Res.* 5 2110–2124.

[B11] Chauhan P. S. Puri N. Sharma P. Gupta N. (2012). Mannanases: microbial sources, production, properties and potential biotechnological applications. *Appl. Microbiol. Biotechnol.* 93 1817–1830. 10.1007/s00253-012-3887-5 22314515

[B12] Ciani M. Comitini F. Mannazzu I. (2008). “Fermentation,” in *Encyclopedia of Ecology*, eds Jørgensen S. E. Fath B. D. (Oxford: Academic Press), 1548–1557.

[B13] Desantis T. Z. Hugenholtz P. Larsen N. Rojas M. Brodie E. L. Keller K. (2006). Greengenes, a chimera-checked 16S rRNA gene database and workbench compatible with ARB. *Appl. Environ. Microbiol.* 72 5069–5072. 10.1128/AEM.03006-05 16820507 PMC1489311

[B14] Dhawan S. Kaur J. (2007). Microbial mannanases: an overview of production and applications. *Crit. Rev. Biotechnol.* 27 197–216. 10.1080/07388550701775919 18085462

[B15] Edgar R. C. (2013). UPARSE: highly accurate OTU sequences from microbial amplicon reads. *Nat. Methods* 10 996–998. 10.1038/nmeth.2604 23955772

[B16] Encarnação J. Abrantes A. Pires A. Botelho M. (2015). Revisit dietary fiber on colorectal cancer: butyrate and its role on prevention and treatment. *Cancer Metastasis Rev.* 34 465–478. 10.1007/s10555-015-9578-9 26224132

[B17] Eom S. H. Park H. J. Jin C. W. Kim D. O. Seo D. W. Jeong Y. H. (2008). Changes in antioxidant activity with temperature and time in *Chrysanthemum indicum* L.(Gamguk) teas during elution processes in hot water. *Food Sci. Biotechnol.* 17 408–412.

[B18] Han Y. Du J. Li J. Li M. (2019). Quantification of the organic acids in hawthorn wine: a comparison of two HPLC methods. *Molecules* 24:2150. 10.3390/molecules24112150 31181607 PMC6600212

[B19] Huang Y. Liu C. Xiao X. (2016). Quality characteristics of a pickled tea processed by submerged fermentation. *Int. J. Food Prop.* 19 1194–1206. 10.1080/10942912.2015.1075217

[B20] Jayabalan R. Marimuthu S. Swaminathan K. (2007). Changes in content of organic acids and tea polyphenols during kombucha tea fermentation. *Food Chem.* 102 392–398. 10.1016/j.foodchem.2006.05.032

[B21] Jeong S. H. Lee S. H. Jung J. Y. Choi E. J. Jeon C. O. (2013). Microbial succession and metabolite changes during long-term storage of Kimchi. *J. Food Sci.* 78 M763–M769. 10.1111/1750-3841.12095 23550842

[B22] Jha A. K. Li J. Yuan Y. Baral N. Ai B. (2014). A review on bio-butyric acid production and its optimization. *Int. J. Agric. Biol.* 16 1019–1024.

[B23] Kanpiengjai A. Chui-Chai N. Chaikaew S. Khanongnuch C. (2016). Distribution of tannin-tolerant yeasts isolated from Miang, a traditional fermented tea leaf (*Camellia sinensis* var. assamica) in northern Thailand. *Int. J. Food Microbiol.* 238 121–131. 10.1016/j.ijfoodmicro.2016.08.044 27614423

[B24] Kawakami M. Chairote G. Kobayashi A. (1987). Flavor constituents of pickled tea, miang, in thailand. *Agric. Biol. Chem.* 51 1683–1687. 10.1271/bbb1961.51.1683

[B25] Khanongnuch C. Asada K. Tsuruga H. Ooi T. Kinoshita S. Lumyong S. (1998). β-Mannanase and xylanase of Bacillus subtilis 5H active for bleaching of crude pulp. *J. Ferment. Bioeng.* 86 461–466. 10.1016/S0922-338X(98)80152-9

[B26] Khanongnuch C. Unban K. Kanpiengjai A. Saenjum C. (2017). Recent research advances and ethno-botanical history of miang, a traditional fermented tea (*Camellia sinensis* var. assamica) of Northern Thailand. *J. Ethn. Foods* 4 135–144. 10.1016/j.jef.2017.08.006

[B27] Kim Y. Goodner K. L. Park J. D. Choi J. Talcott S. T. (2011). Changes in antioxidant phytochemicals and volatile composition of *Camellia sinensis* by oxidation during tea fermentation. *Food Chem.* 129 1331–1342. 10.1016/j.foodchem.2011.05.012

[B28] Kõljalg U. Nilsson R. H. Abarenkov K. Tedersoo L. Taylor A. F. Bahram M. (2013). Towards a unified paradigm for sequence-based identification of fungi. *Mol. Ecol.* 22 5271–5277. 10.1111/mec.12481 24112409

[B29] Kosińska A. Andlauer W. (2014). “Antioxidant capacity of tea: effect of processing and storage,” in *Processing and Impact on Antioxidants in Beverages*, Ed. Preedy V. (Amsterdam: Elsevier), 109–120. 10.1016/b978-0-12-404738-9.00012-x

[B30] Li Q. Huang J. Li Y. Zhang Y. Luo Y. Chen Y. (2017). Fungal community succession and major components change during manufacturing process of Fu brick tea. *Sci. Rep.* 7 1–9. 28761046 10.1038/s41598-017-07098-8 PMC5537287

[B31] Li Z. Feng C. Luo X. Yao H. Zhang D. Zhang T. (2018). Revealing the influence of microbiota on the quality of Pu-erh tea during fermentation process by shotgun metagenomic and metabolomic analysis. *Food Microbiol.* 76 405–415. 10.1016/j.fm.2018.07.001 30166168

[B32] Liang H. Chen H. Ji C. Lin X. Zhang W. Li L. (2018). Dynamic and functional characteristics of predominant species in industrial paocai as revealed by combined DGGE and metagenomic sequencing. *Front. Microbiol.* 9:2416. 10.3389/fmicb.2018.02416 30356774 PMC6189446

[B33] Loman N. J. Misra R. V. Dallman T. J. Constantinidou C. Gharbia S. E. Wain J. (2012). Performance comparison of benchtop high-throughput sequencing platforms. *Nat. Biotechnol.* 30 434–349. 10.1038/nbt.2198 22522955

[B34] Magoč T. Salzberg S. L. (2011). FLASH: fast length adjustment of short reads to improve genome assemblies. *Bioinformatics* 27 2957–2963. 10.1093/bioinformatics/btr507 21903629 PMC3198573

[B35] Makkar H. P. Blümmel M. Borowy N. K. Becker K. (1993). Gravimetric determination of tannins and their correlations with chemical and protein precipitation methods. *J. Sci. Food Agric.* 61 161–165. 10.1002/jsfa.2740610205

[B36] Mcdougall G. J. Morrison I. M. Stewart D. Weyers J. D. B. Hillman J. R. (1993). Plant fibres: botany, chemistry and processing for industrial use. *J. Sci. Food Agric.* 62 1–20. 10.1002/jsfa.2740620102

[B37] Miller G. L. (1959). Use of dinitrosalicylic acid reagent for determination of reducing sugar. *Anal. Chem.* 31 426–428. 10.1021/ac60147a030

[B38] Mugula J. K. Narvhus J. A. Sørhaug T. (2003). Use of starter cultures of lactic acid bacteria and yeasts in the preparation of togwa, a Tanzanian fermented food. *Int. J. Food Microbiol.* 83 307–318. 10.1016/S0168-1605(02)00386-0 12745235

[B39] Narvhus J. A. Gadaga T. H. (2003). The role of interaction between yeasts and lactic acid bacteria in African fermented milks: a review. *Int. J. Food Microbiol.* 86 51–60. 10.1016/S0168-1605(03)00247-2 12892921

[B40] Neffe-Skocińska K. Sionek B. Ścibisz I. Kołożyn-Krajewska D. (2017). Acid contents and the effect of fermentation condition of Kombucha tea beverages on physicochemical, microbiological and sensory properties. *CyTA J. Food* 15 601–607. 10.1080/19476337.2017.1321588

[B41] Noh D. O. Choi H. S. Suh H. J. (2014). Catechine biotransformation by tannase with sequential addition of substrate. *Process Biochem.* 49 271–276. 10.1016/j.procbio.2013.11.001

[B42] Okada S. Daengsubha W. Uchimura T. Ohara N. Kozaki M. (1986). Flora of lactic acid bacteria in miang produced in northern Thailand. *J. Gen. Appl. Microbiol.* 32 57–65. 10.2323/jgam.32.57

[B43] Olanbiwoninu A. A. Odunfa S. A. (2018). Microbial interaction in selected fermented vegetable condiments in Nigeria. *Int. Food Res. J.* 25 439–455.

[B44] Pou K. R. J. (2016). Fermentation: the key step in the processing of black tea. *J. Biosyst. Eng.* 41 85–92. 10.5307/JBE.2016.41.2.085

[B45] Rahman A. Hayati N. Chieng B. W. Lbrahim N. A. Abdul Rahman N. (2017). Extraction and characterization of cellulose nanocrystals from tea leaf waste fibers. *Polymers* 9:588. 10.3390/polym9110588 30965890 PMC6418996

[B46] Rephaeli A. Zhuk R. Nudelman A. (2000). Prodrugs of butyric acid from bench to bedside: synthetic design, mechanisms of action, and clinical applications. *Drug Dev. Res.* 50 379–391. 10.1002/1098-2299(200007/08)50:3/4<379:AID-DDR20<3.0.CO;2-8

[B47] Santana M. A. Moccia-V C. C. Gillis A. (2008). Bacillus thuringiensis improved isolation methodology from soil samples. *J. Microbiol. Methods* 75 357–358. 10.1016/j.mimet.2008.06.008 18619500

[B48] Shannon C. E. Weaver W. (1963). *A Mathematical Theory of Communication.* Illinois: University of Illinois Press.

[B49] Sharma H. S. S. Faughey G. Lyons G. (1999). Comparison of physical, chemical, and thermal characteristics of water-, dew-, and enzyme-retted flax fibers. *J. Appl. Polym. Sci.* 74 139–143. 10.1002/(SICI)1097-4628(19991003)74:1<139:AID-APP17<3.0.CO;2-E

[B50] Sukontasing S. Tanasupawat S. Moonmangmee S. Lee J. S. Suzuki K. I. (2007). *Enterococcus camelliae* sp. nov., isolated from fermented tea leaves in Thailand. *Int. J. Syst. Evol. Microbiol.* 57 2151–2154. 10.1099/ijs.0.65109-0 17766890

[B51] Tan G. Hu M. Li X. Pan Z. Li M. Li L. (2020). High-throughput sequencing and metabolomics reveal differences in bacterial diversity and metabolites between red and white Sufu. *Front. Microbiol.* 11:758. 10.3389/fmicb.2020.00758 32390991 PMC7188790

[B52] Tanasupawat S. Pakdeeto A. Thawai C. Yukphan P. Okada S. (2007). Identification of lactic acid bacteria from fermented tea leaves (miang) in Thailand and proposals of *Lactobacillus thailandensis* sp. nov. *Lactobacillus camelliae* sp. nov., and *Pediococcus siamensis* sp. nov. *J. Gen. Appl. Microbiol.* 53 7–15. 10.2323/jgam.53.7 17429157

[B53] Tasev K. Stefova M. Ivanova V. (2016). HPLC method validation and application for organic acid analysis in wine after solid-phase extraction. *Maced. J. Chem. Chem. Eng.* 35 225–233.

[B54] Unban K. Khatthongngam N. Shetty K. Khanongnuch C. (2019). Nutritional biotransformation in traditional fermented tea (Miang) from north Thailand and its impact on antioxidant and antimicrobial activities. *J. Food Sci. Technol.* 56 2687–2699. 10.1007/s13197-019-03758-x 31168151 PMC6525707

[B55] Vina I. Semjonovs P. Linde R. Patetko A. (2013). Glucuronic acid containing fermented functional beverages produced by natural yeasts and bacteria associations. *Int. J. Res. Rev. Appl. Sci.* 14 17–25.

[B56] Xiao P. Huang Y. Yang W. Zhang B. Quan X. (2015). Screening lactic acid bacteria with high yielding-acid capacity from pickled tea for their potential uses of inoculating to ferment tea products. *J. Food Sci. Technol.* 52 6727–6734. 10.1007/s13197-015-1803-6 26396422 PMC4573110

[B57] Zhang J. He Z. Hu K. (2000). Purification and characterization of β-mannanase from Bacillus licheniformis for industrial use. *Biotechnol. Lett.* 22 1375–1378. 10.1023/A:1005644414762

[B58] Zhu M. Z. Li N. Zhou F. Ouyang J. Lu D. M. Xu W. (2020). Microbial bioconversion of the chemical components in dark tea. *Food Chem.* 312:126043. 10.1016/j.foodchem.2019.126043 31896450

